# Clinical Lectures on Mental Diseases

**Published:** 1899-09

**Authors:** 


**Clinical Lectures on Mental Diseases.** By T. S. Clouston, M.D. Fifth Edition. Pp. xii., 727. London: J. & A. Churchill. 1898.

We know of no book which better deserves its popularity than this one. Designed as a clinical manual for students of mental diseases and general practitioners, the book forbears intimate discussion on many vexed questions, and hence does not invite, in greater part, the criticism which awaits the propounder of hypotheses. The author has advanced, almost of necessity, certain assumptions which may invite discussion amongst his fellow-alienists; but it would be straining the function of criticism to raise this here whilst there is so much matter of the highest value to receive comment; it should be said, that to no debatable opinions of his own does Dr. Clouston give undue or misleading prominence.

There is evidence in this work of acute observation, large and shrewd views of sane as of insane humanity, unsurpassed experience and versatility of treatment. In our opinion no modern physician has better conceived and practised, in its widest sense, the treatment of insanity; from the aspect of therapeusis alone the book is of great worth. Exceptional, too, are the powers of graphic description, of harmonious groupings of varied clinical pictures, and of sound generalisation, and as the result there is aroused a vivid interest in the cases described. Of especial utility will be found the sections relating to the examination and certification of persons of unsound mind, together with the various contingencies arising in connection with these procedures.

This edition has received important addition in the excellent plates and descriptive letter-press which demonstrate some most recent processes in microscopic technique. In short, anyone who digests this book will have acquired a valuable knowledge of the clinical aspects of insanity, and despite the endless variety of these will always find typical descriptions, ripe experience, and happy suggestions to guide him.

